# Use of cues in virtual reality depends on visual feedback

**DOI:** 10.1038/s41598-017-16161-3

**Published:** 2017-11-22

**Authors:** Jacqueline M. Fulvio, Bas Rokers

**Affiliations:** Department of Psychology, McPherson Eye Research Institute University of Wisconsin – Madison, Madison, USA

## Abstract

3D motion perception is of central importance to daily life. However, when tested in laboratory settings, sensitivity to 3D motion signals is found to be poor, leading to the view that heuristics and prior assumptions are critical for 3D motion perception. Here we explore an alternative: sensitivity to 3D motion signals is context-dependent and must be learned based on explicit visual feedback in novel environments. The need for action-contingent visual feedback is well-established in the developmental literature. For example, young kittens that are passively moved through an environment, but unable to move through it themselves, fail to develop accurate depth perception. We find that these principles also obtain in adult human perception. Observers that do not experience visual consequences of their actions fail to develop accurate 3D motion perception in a virtual reality environment, even after prolonged exposure. By contrast, observers that experience the consequences of their actions improve performance based on available sensory cues to 3D motion. Specifically, we find that observers learn to exploit the small motion parallax cues provided by head jitter. Our findings advance understanding of human 3D motion processing and form a foundation for future study of perception in virtual and natural 3D environments.

## Introduction

The perception of 3D motion is fundamental to our interactions with the environment, but we have poor insight into the sensory cues that support its accuracy. Previous studies have reported poor sensitivity to 3D motion cues^1^, and some work has reported that observers will discount 3D motion cues altogether^2^. Poor sensory sensitivity will inevitably lead to inconsistent behavioral performance, but may also lead to more systematic perceptual errors. Such errors have been reported in the judgment of 3D motion. Observers will judge that approaching objects will miss the head, even when they are on a collision course^3,4^. Observers will also judge approaching objects to be receding and vice versa^5^. This has led to the view that 3D motion perception relies in large part on heuristics and prior assumptions^6^.

Here we consider another possibility: that the sensitivity to 3D motion cues is context-dependent and needs to be learned for a given visual environment based on explicit visual feedback. Virtual reality (VR) provides the ideal tool to investigate sensitivity to 3D motion because it allows us to present sensory signals that closely approximate those in the real world, while maintaining tight experimental control. We manipulated the sensory cues thought to contribute to perception in VR environments and tested the role of experience and feedback on performance.

We first explored the extent to which the virtual environment supported accurate sensory processing of 3D motion information “out of the box”. We asked observers to intercept targets that moved in a 3D environment, and found performance to be generally poor. One of the compelling features of VR-based viewing is that it can provide motion parallax cues, i.e., head-motion contingent updating of the visual display. Such cues are not available in most traditional visual experiments. We found that the addition of motion parallax cues produced by small naturally-occurring random head-motion (head jitter) did not improve performance. Observers were insensitive to the additional cues even after prolonged exposure to the stimuli. This result is consistent with the notion that head jitter-based cues are too small, or too noisy to have a meaningful impact.

Second, we tested the hypothesis that observers require explicit visual feedback when immersed in a new (virtual) environment. Consistent with feedback-driven sensory recalibration^7–11^, rapid and significant improvements in performance were observed when visual feedback was provided following the observer’s actions. In particular, the cues to motion and depth provided by head jitter that had no effect in the first part of the study became an important source of sensory information.

In summary, we identified the sensory cues that contribute to 3D motion perception and the conditions under which they are employed. Our results advance understanding of human visual processing in 3D environments. Furthermore, these results help explain the varying levels of success 3D and virtual reality displays have enjoyed both in research and entertainment settings and suggest best practices in using VR technology.

## Results

**Figure 1:**
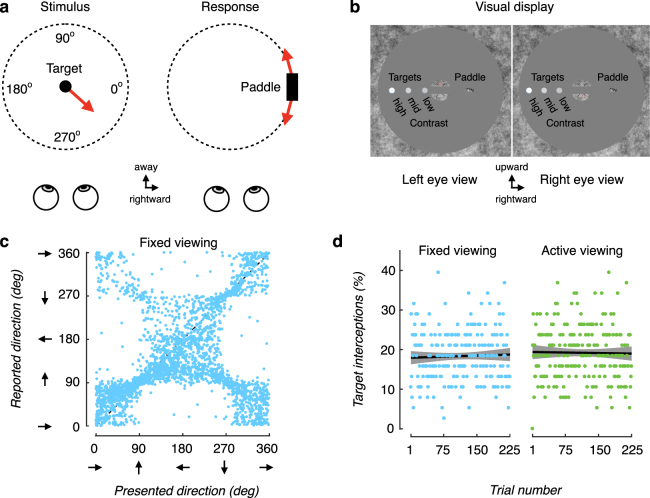
Performance on a 3D motion perception task, and the impact of active viewing. (**a**) *Experimental procedure without feedback*. Observers judged the 3D direction of a moving dot (target). The target appeared in the center of the display at the start of a trial. After the target traversed a randomly chosen trajectory (‘Presented direction’), it disappeared and a response ‘paddle’ appeared. Observers adjusted the position of the paddle around a circular orbit so that it would have intercepted the target had it continued along its trajectory (‘Reported direction’). No feedback was provided. (**b**) Illustration of left- and right-eye stimulus elements as presented in the experiments. (**c**) Reported motion direction as a function of presented direction for all 38 observers (75 trials each, targets presented at mid contrast under the fixed viewing condition). Data points along the positive diagonal correspond to accurate reports of 3D motion trajectory. Deviations from that diagonal correspond to misreports of motion trajectories. (**d**) Performance (% of target interceptions) as a function of trial number. An interception was defined as a reported target direction that would hit the paddle (within +/− 8 deg of the presented direction). % Interceptions did not differ significantly between the fixed viewing condition (blue symbols) and active viewing (motion parallax) condition (green symbols), either at the beginning of the experiment, or with repeated exposure. Data points correspond to the percentage of target interceptions across observers on each trial; solid lines correspond to the exponential fit to the data, and the shaded regions correspond to the 95% confidence intervals on the exponential fit.

To identify the roles of head movement and task feedback on perceptual accuracy, 38 observers performed a 3D motion extrapolation task (‘3D Pong’) in a virtual reality environment. We used the Oculus Rift Development Kit 2 (DK2) head-mounted display, which provides low-latency translation and rotation (6 degrees of freedom) head-tracking (www.oculusvr.com). On each trial, a white sphere (‘target’) appeared at the fixation point and moved in a random direction in x- (lateral) and z- (depth) with no change in y (vertical) within the 360 deg space for 1 s before disappearing (Fig. 1a). Observers moved a rectangular block (‘paddle’) along an invisible orbit centered on the target origin and were asked to adjust the paddle position such that it would have intercepted the target had it continued along the trajectory. A version of the task using 3D shutter glasses rather than full immersive VR has been described previously^5^.

Observers participated in three randomized blocks of trials that differed according to the impact of small random head movements (jitter) on scene rendering: (i) ‘fixed’ – in which the scene did not update with head position; (ii) ‘active’ – in which the scene immediately updated according to head position; (iii) and a ‘lagged’ control condition – in which the scene either updated in response to head position with a 50 ms delay (beyond the inherent latency of the system - see **Methods** for details) or with a 0–500 ms delay chosen randomly from a uniform distribution on each trial. Additionally, target contrast was randomly chosen from one of three levels (low - 0.47; mid - 0.55; high - 0.75 Weber fraction).

### Performance in a head-fixed VR environment

We first assessed performance in the fixed condition to obtain a performance baseline. Performance was characterized by three key features. First, observers tended to misreport 3D motion direction such that some approaching targets were judged to be receding and vice versa (revealed by the data points falling along the negative diagonal in Fig. 1c). Second, the prevalence of these misreports decreased monotonically with increases in target contrast (F(2,74) = 15.25, p < 0.001, η^2^ = 0.29, repeated-measures ANOVA). Third, approaching targets were more likely to be reported as receding than vice versa (revealed by the heavier mass along the negative diagonal on the lower half of the plot in Fig. 1c). This pattern of results is in line with our prior findings using 3D (shutter) glasses under head-fixed viewing conditions^5^ and is consistent with poor sensitivity to sensory cues that specify 3D motion^1–6^.

### Performance in an active VR environment

Accurate 3D motion perception may critically depend on sensory cues that are not typically available in traditional visual displays. We therefore wanted to assess performance in an active viewing condition where motion parallax cues were made available. In this condition, the visual scene updated in accordance with the observer’s small random head motions (jitter) and the laws of perspective projection. We found that the addition of head jitter-based cues did not improve perceptual accuracy. Performance did not differ between the active and fixed conditions (two-factor repeated-measures ANOVA, p = 0.86) for all levels of target contrast (p > 0.05 for all three paired-sample t-tests). Thus, the availability of sensory cues in a viewing environment did not imply their usage. Furthermore, the results provide no evidence that observers adjusted to the new viewing environment over time - their performance did not improve following considerable exposure to the stimuli (Fig. 1c).

It is possible that the head jitter-based cues are too small, too noisy, or too short a duration to impact 3D motion perception. For example, head jitter may contribute noise by reducing the precision of fixation^12^. As such, one might expect the visual system to down-weight the noisy information in order to maintain stable performance. Such an explanation could account for the results above, which demonstrated equivalent levels of performance in the fixed and active conditions (no main effects of target contrast or viewing condition on performance, (p = 0.77, p = 0.74), respectively, nor an interaction between the two (p = 0.26) in a two-factor repeated-measures ANOVA). We will return to this issue in a subsequent section.

### Role of task feedback

**Figure 2:**
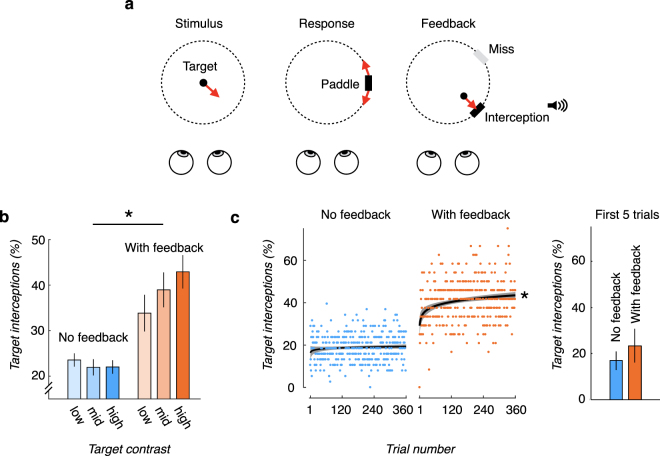
Task feedback leads to rapid improvement in 3D motion perception. (**a**) *Experimental procedure with feedback*. Observers first viewed a target stimulus and responded by adjusting the location of a paddle so that it would intercept the target. During the feedback stage, the target would reappear and continue along its trajectory until it intersected with the paddle’s orbit, and an auditory signal would indicate an interception or a miss. (**b**) *Task feedback increased target interceptions at all contrast levels*. Target interceptions during fixed viewing without (blue bars) and with (orange bars) feedback for the three target contrast levels. * corresponds to a significant difference between conditions (p < 0.05). (**c**) *Task feedback improves performance over time*. Left: Target interceptions over trials when feedback was not provided (blue symbols) and when feedback was provided (orange symbols). Circles correspond to the percentage of target interceptions across observers on each trial; solid lines correspond to the exponential fit to the data (circles), and the shaded regions correspond to the 95% confidence intervals on the exponential fit. * indicates a significant slope (p < 0.05). Right: Mean performance for the no feedback and feedback groups during the first 5 trials. The difference between the groups is not significant. Error bars correspond to +/− 1 SEM.

The results so far cannot distinguish whether the sensory cues in these simulated environments are not useful or whether observers do not take advantage of them. Additionally, the impact of cue conflicts in the simulated environments, i.e., between the simulated depths of the objects in the virtual display and “flatness cues” such as accommodation^13,14^, is unclear. Unlike in the experiments described above, behavioral feedback about one’s performance is often available in real world tasks, and experimental evidence suggests that such feedback may play a key role in learning and improvement in performance on a variety of visuomotor tasks^15–24^. For example, feedback may signal the validity of available sensory information for the task (e.g.^25^). To test the hypothesis that explicit behavioral feedback encourages the recruitment of sensory cues, we modified the 3D Pong task such that after observers provided their response by locking in their paddle setting, the target reappeared at its last location and continued along its trajectory until it intersected the paddle’s orbit (Fig. 2a). If the setting was correct, the paddle would intercept the target and a hit tone would play; otherwise, the target would miss the paddle and a ‘swoosh’ tone would play. We found that the presence of visual task feedback substantially improved target interceptions, when compared to performance in the absence of task feedback (F(1,180) = 8.78, p < 0.01; Fig. 2b). The improvement occurred for all three target contrast levels (p < 0.001 with Bonferroni-corrected alpha-level = 0.0167 for multiple comparisons), and was significantly larger for high contrast targets (+20% improvement), than for mid (+17%) or low (+10%) contrast targets (F(2,46) = 7.82, p < 0.01, repeated-measures ANOVA). Improvements were such that performance in the lowest contrast condition with feedback (42.9% interceptions) was considerably better than performance in the highest target contrast condition without feedback (23.6% interceptions).

### Role of head jitter

Our previous results showed no benefit of head jitter on performance. However, it may be that observers require explicit task feedback to learn and take advantage of head jitter-contingent motion parallax cues.

**Figure 3:**
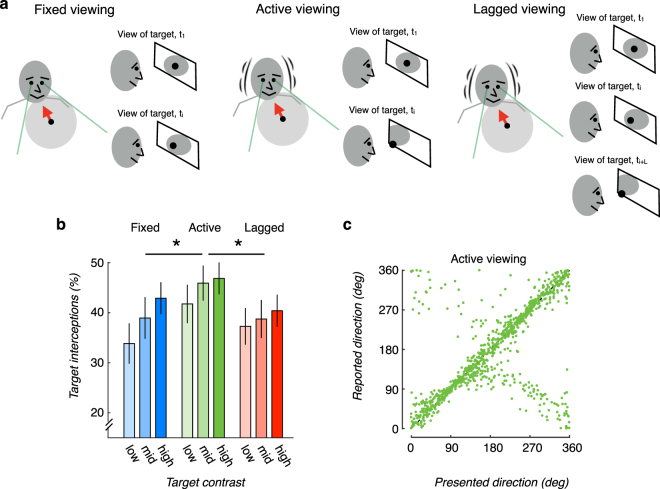
Impact of head jitter on performance when task feedback is provided. (**a**) *Schematic comparing the three viewing conditions*. In fixed viewing (left schematic), the scene does not update in response to head jitter, thereby leaving the viewpoint from which the observers view the stimuli constant for the duration of the experimental block, independent of any observer head motion. In active viewing (middle schematic), the visual scene updates in response to head jitter, thus (slightly) changing the viewpoint from which the observers observe the stimuli. In lagged viewing (right schematic), the scene updates with a random 0–500 ms delay (denoted ‘*L’* in the figure) beyond the inherent latency of the VR system. Note that the lagged updating does not preclude the trial events from occurring at their normal pace. (**b**) *Head jitter contingent motion parallax improves performance*. Percentage of trials in which observers’ paddle settings intercepted the target for each of the three viewing conditions and target contrast levels. Error bars correspond to +/− 1 SEM. A * corresponds to a significant paired-sample difference between the two viewing conditions at the two-tailed Bonferroni-corrected alpha level = 0.0167 for multiple comparisons. (**c**) *Motion parallax reduces perceptual biases and errors*. Reported motion direction as a function of presented direction for all 24 observers (40 trials each, targets presented at mid contrast, under the active viewing condition with task feedback) plotted for comparison with Fig. 1c.

Indeed, performance improved when observers received behavioral feedback, indicating that feedback plays a critical role in the recruitment of the available sensory cues. A two-factor repeated-measures ANOVA comparing performance across all three viewing conditions revealed a significant main effect of viewing condition when feedback was provided (F(2,184) = 3.67, p = 0.033). Critically, a follow-up comparison investigating the individual effects of viewing condition revealed a significant difference in performance between the fixed and active viewing conditions. The percentage of target interceptions in the active viewing condition was larger than performance in the fixed condition (*M*_*active*_ = *44*.*9%*, *M*_*fixed*_ = *38*.*6%;* t(71) = 3.95, p < 0.001, d = 0.47, paired-sample t-test at the Bonferroni-corrected alpha level = 0.0167 for multiple comparisons - additional tests will be described below). We note that observers spent a relatively short time in each condition. Behavioral performance in the feedback condition did not yet reach asymptotic levels in the feedback condition (Fig. 2c). We speculate that additional time spent training with feedback would magnify the impact of head jitter on perceptual accuracy. Finally, the main effect of target contrast was robust in the presence of feedback (F(2,184) = 7.89, p = 0.001) and no evidence for an interaction between viewing condition and target contrast was found (F(4,184) = 1.31, p = 0.27; Fig. 3b).

The improvement in performance can be appreciated by comparing the raw data for the active condition with visual and auditory feedback plotted in Fig. 3c with the raw data for the fixed condition without feedback plotted in Fig. 1c. Visual inspection of these two plots reveals that the presence of feedback results in far fewer reversals (i.e., a much diminished negative diagonal in Fig. 3c) and a much tighter clustering of data points around the positive diagonal, especially around the leftward (180 deg) and rightward (0 deg) presented directions. Given that the trajectories are determined by combinations of independent and random speeds in the lateral and in-depth directions, this suggests that feedback improves sensitivity to binocular information more generally, rather than specifically to either motion direction.

To verify that improvements in the active viewing condition were due to the use of motion parallax cues, we wanted to exclude other possibilities. Head jitter contingent updating of the visual display effectively increases the display’s spatial resolution. We therefore examined performance in a lagged viewing condition that reduced the reliability of the motion parallax cues, but produced the same improvement in effective spatial resolution (Fig. 3a). Performance differed significantly between the active and lagged viewing conditions. The percentage of target interceptions in the active viewing condition was significantly larger than in the lagged viewing condition (*M*_*active*_ = *44*.*9%*, *M*_*lagged*_ = *38*.*8%;* t(71) = 3.51, p < 0.001, d = 0.41, paired-sample t-test at the Bonferroni-corrected alpha level = 0.0167 for multiple comparisons). In fact, performance in the lagged viewing condition was comparable to performance in the fixed viewing condition (*M*_*fixed*_ = *38*.*6%*, *M*_*lagged*_ = *38*.*8%;* t(71) = 0.12, p = 0.9, d = 0.01, paired-sample t-test at the Bonferroni-corrected alpha level = 0.0167 for multiple comparisons). These results suggest that the motion parallax information in the active viewing condition is a reliable source of motion-in-depth information that observers can (and do) learn to take advantage of.

We next measured improvements over time for each of the three individual viewing conditions when feedback was provided. We found significant improvements in performance from the first trial to the last in the active viewing condition (exponential fit, b = 0.0015, 95% CI: [0.0004, 0.0026], p < 0.01) and in the lagged condition (b = 0.002, 95% CI: [0.0006, 0.0033], p < 0.01). The change in performance in the fixed condition was also positive, but did not reach significance (b = 0.001, 95% CI: [−0.0002, 0.0024]). By contrast, when task feedback was not provided, we found no significant change over time in any of the conditions.

We note that the improvements observed in all three conditions when visual and auditory feedback were provided (Fig. 3b) may have been due, in part, to transfer of feedback-based learning across experimental blocks. In particular, the superior performance in the active condition suggests that the greatest benefits of task feedback were derived in this condition. Because each observer participated in the fixed, active, and lagged conditions in a randomized order, for 67% of observers the fixed and lagged conditions were preceded by experience in the active condition. This experience may have thus transferred to benefit performance in those blocks. To address this, we analyzed performance in the fixed and lagged conditions with feedback, broken down into two groups: 1. when the observer experienced the active condition in a previous block; 2. when the observer experienced the active condition in a subsequent block (results not shown). The analysis revealed that there was no difference in performance in either the fixed or lagged viewing condition whether it was preceded by or followed by the active viewing condition (p = 0.77 (fixed); p = 0.35 (lagged)). This demonstrates that the presence of task feedback significantly improved performance in all three viewing conditions, which cannot be attributed solely to transfer from the active viewing condition.

Additionally, comparing the trial-to-trial trends from the first trial that each observer experienced to the last, independent of viewing condition, it is evident that observers who received feedback initially performed at a level similar to that of the observers who did not receive feedback within the first 5 trials (p = 0.12; 23.3% (+/−16.3%) vs 16.8% (+/−9.1%)) and rapidly improved (Fig. 2c). The steady improvement over time further demonstrates a continued benefit of feedback above and beyond any transfer of previous experience.

Presence of visual feedback necessarily implied longer exposure to target motion. We wished to address the possibility that the behavioral improvements were due to feedback per se rather than the longer exposure. We therefore conducted an additional analysis in which we equated the viewing time across the two groups. For the no feedback conditions, observers viewed 225 1-second trials per viewing condition (fixed, active, lagged). We therefore compared performance to the first 225 seconds of presented target motion in the feedback group, which included both the 1 s stimulus trajectories, and the target motion during the visual feedback interval. For the feedback conditions, this corresponded to an average of about 39 trials per viewing condition. When equating the viewing time in this way, we still found superior performance in the feedback conditions compared to the no feedback conditions (F(1,180) = 200.23, p < 0.001), ruling out an explanation based on mere exposure to target motion.

**Figure 4:**
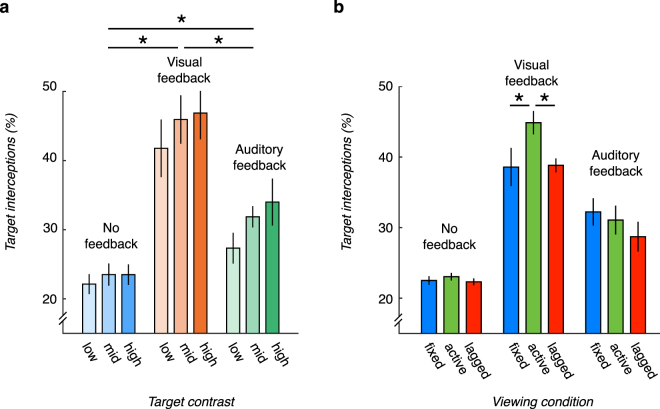
Benefit of visual feedback over auditory and no feedback in 3D motion perception. (**a**) *Visual task feedback confers a superior benefit over auditory-only feedback in active viewing*. Target interceptions during active viewing without feedback (left bars), with visual feedback (middle bars), and with auditory-only feedback (right bars) for the three target contrast levels. Error bars correspond to +/− 1 SEM. A * corresponds to a significant difference between the feedback conditions (p < 0.025, Bonferroni-corrected). (**b**) *Head jitter-contingent motion parallax improves performance when visual feedback is present*. Percentage of target interceptions for each of the three feedback and viewing conditions. The combination of visual feedback and active viewing produces superior accuracy in 3D motion perception. Error bars correspond to +/− 1 SEM. * corresponds to a significant different between the two viewing conditions (p < 0.0167, Bonferroni-corrected).

Finally, we asked whether visual, rather than auditory, feedback is required for improvements in performance. We measured performance in an auditory-only task feedback condition in which a hit or miss tone played on each trial, but no visual indication of the error in the observer’s paddle setting location was provided. In general, manipulating task feedback mattered for performance (F(2,207) = 18.33, p < 0.001; Fig. 4a). Performance with auditory-only feedback was significantly better than when no feedback was provided ((F(1,138) = 62.35, p < 0.001). However, the benefit of auditory-only feedback fell well short of combined auditory and visual feedback (F(1,96) = 53.03, p < 0.001), even with the larger number of trials that observers experienced in the auditory-only feedback condition. Furthermore, while auditory-only feedback improved performance in all three viewing conditions (i.e., fixed, active, and lagged) relative to when feedback was not provided, no differences in performance were observed across the three viewing conditions (p = 0.19, Fig. 4b). Moreover, only in the fixed auditory-only feedback condition did the improvement in performance reach the level of performance in the visual feedback condition (difference between fixed auditory-only and fixed visual feedback with *p* = 0.08, and significant differences between performance in the auditory-only and visual feedback in the active and lagged conditions with *p* < 0.01). These results suggest that auditory feedback is preferable over no feedback in helping observers carry out tasks in novel virtual environments, but visual feedback appears to be most effective in improving observer performance.

We emphasize that head motion information was available throughout the entire experiment, not just during the feedback stage. As such, observers in the no feedback group and the auditory-only feedback group also had access to the information. Combined with the different levels of performance among the fixed, active, and lagged conditions in the visual + auditory feedback condition, these results reveal the importance of visual feedback in training. These results however, leave open the question what exactly is learned when feedback is provided. We address this question in the next section.

### Quantification of head jitter

The performance of untrained observers suggests that they do not take advantage of all available cues to depth in a virtual reality environment. We have shown that feedback significantly improves perceptual performance. However, the question of what exactly the observers are learning based on the visual feedback remains.

**Figure 5:**
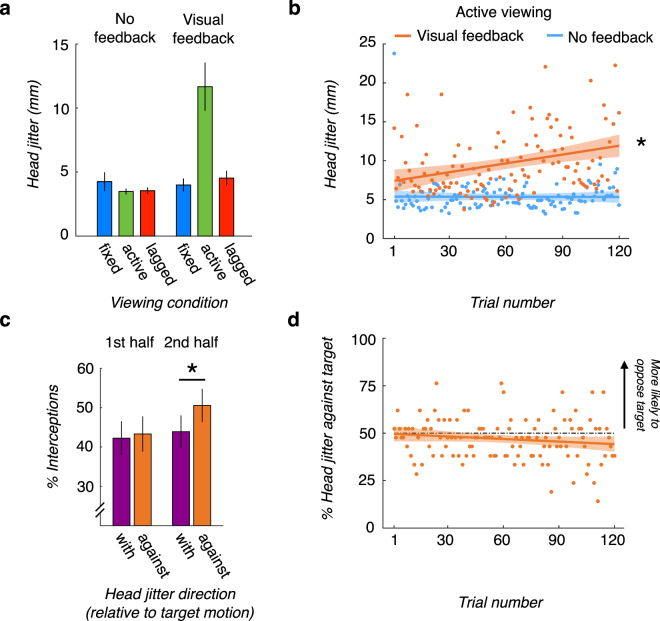
Quantification of head jitter. (**a**) *Head jitter is suppressed under conditions in which observers do not rely upon motion parallax information*. Head jitter during the one-second target motion intervals without feedback (left bars), and with visual feedback (right bars) for the three viewing conditions. Error bars correspond to +/− 1 SEM. The head displacement in the active condition with feedback is significantly larger than all other conditions (*p* < 0.001 with Bonferroni-corrected alpha level = 0.01). (**b**) *Head jitter gradually increases over time*. Head jitter in the active viewing condition with visual feedback (orange symbols) as a function of trial to be compared to with head jitter in the active viewing condition without feedback (blue symbols). Circular symbols correspond to the between-subject median head jitter on each trial, the solid lines correspond to the linear fit to the symbols, and the shaded regions correspond to the 95% confidence intervals on the linear fit. *denotes a slope significantly greater than zero at the alpha = 0.05 level. (**c**) *Head jitter that opposes the target stimulus direction is more likely to lead to a target interception during the second half of the experimental block*. Probability of target interception as a function of head jitter direction relative to the target’s motion in the active viewing condition with visual feedback during the first half of trials (left bars) and the second half of trials (right bars). *denotes a significant difference at the Bonferroni-corrected alpha level = 0.025. (**d**) *The increase in head jitter does not reflect a particular movement strategy*. Probability of head jitter opposing the target motion direction as a function of trial in the active viewing condition with feedback. Orange symbols correspond to the between-subject mean probability, the solid orange line corresponds to the linear fit to the symbols, the shaded region corresponds to the 95% confidence interval on the linear fit, and the black dashed line corresponds to the absence of a strategy at 50%. The slope of the line is not statistically different from 50%.

To investigate the potential impact of feedback on head jitter, we quantified the magnitude of head jitter as observers viewed the target’s 1-second motion trajectory on every trial in each of the experimental conditions. The Oculus Rift provides positional head-tracking with submillimeter accuracy (0.05 mm precision). On average, head jitter was small, at 5.24 mm on average across all conditions. However, the magnitude of head jitter was significantly larger in the active condition when visual task feedback was provided in comparison to all other experimental conditions (*p* < 0.001 for all five comparisons with Bonferroni corrected alpha = 0.01; Fig. 5a).

Importantly, we found that this pattern developed over time. At the onset of the active viewing with visual feedback block, head jitter was comparable to that in other conditions (p > 0.05). However, over the course of the experimental block head jitter increased gradually (b = 0.038, 95% CI: [0.017, 0.059]; Fig. 5b).

We subsequently characterized the relationship between the net direction of head jitter and behavioral performance. By the second half of the experimental block, target interceptions were more likely when head jitter direction was opposite target motion direction (*t(20)* = *2*.*86*, *p* < 0.01; Fig. 5c). However, the behavioral improvement did not seem due to the adoption of a conscious strategy. Observers exhibited no bias to move either with or against the target’s direction at the beginning of the experimental block. Importantly, they remained no more likely to move in the direction opposite the target’s motion by the end of the experimental block (b = −0.045, 95% CI: [−0.103, 0.012]; Fig. 5d).

These results suggest that visual feedback provides important signals about the availability and reliability of sensory cues in a virtual environment. While observers were aware of their accuracy on a trial to trial basis, they subjectively reported that they did not move their head during the task under any of the viewing conditions. Thus, even though the observer was unaware of it, when visual feedback was present, head jitter increased over time and improved perceptual performance based on the available signals – a hallmark of implicit learning.

Why was head jitter so much smaller in all other conditions, including the active viewing – no feedback condition where head-motion contingent motion parallax cues would have been informative? We hypothesize that observers typically suppress head motion in standard experimental displays. In most 2D and 3D displays head motion will introduce cue conflicts - movement of the head does not lead to a corresponding change in the visual scene. Consequently, head motion under such conditions is a source of sensory noise, rather than a potentially reliable and useful source of sensory information. Visual feedback can serve to relax the suppression of head motion in the context of virtual reality displays.

The visual feedback implicitly encouraged observers to move their head freely in a manner more similar to natural scene viewing, as opposed to watching a scene on a TV or computer screen. That observers were not aware of the increase in head movement, yet were able to benefit from it suggests that the contributions of head jitter to the improved performance observed in the 3D Pong task were opportunistic – when observers moved in the direction that opposed the target’s motion direction, they performed better, but this only occurred when they happened to do so. Thus, head jitter changes in a context-dependent manner, and its usefulness needs to be learned for a given visual environment based on explicit visual feedback.

## Discussion

In the current study, we used a ‘gamified’ experimental task (‘3D Pong’) in a virtual reality (VR) environment to assess the factors that contribute to accurate 3D motion perception. We found that when head-tracking capabilities were turned off and task feedback was withheld, performance was similar to that in more traditional 3D experiments using Wheatstone stereoscopes or 3D shutter glasses. Specifically, observers frequently misreported the direction of object motion indicating approaching targets as receding and vice versa. These misreports became more prevalent with decreases in target contrast. Adding motion parallax cues did not improve performance as might be expected from a cue combination standpoint. Such results would support the view that our sensitivity to cues that signal 3D motion is relatively weak.

We then explored an alternative hypothesis, that learning to recruit all relevant and reliable cues in a novel (VR) environment requires training with explicit task feedback. When observers were tested on the same task, but were given explicit task feedback after their response, observers showed significant improvements in performance and significant increases in the magnitude of head jitter. Furthermore, the presence of visual feedback was associated with significant improvements in performance based on the availability of motion parallax cues, but only when the head motion cues were reliable - delays in the corresponding visual information with head motion negated any improvement or changes in head jitter magnitude. Thus, it is not that our sensitivity to cues related to 3D motion is weak per se, but rather, that learning to rely on such cues is a critical component of accurate perception in virtual 3D environments.

The role of feedback in performance has been mixed in the literature. Some previous results suggest that feedback may not be necessary to improve task performance (e.g.^26^), with numerous reports of successful learning with no external feedback^18,27–31^. In fact, some studies have shown that perceptual learning may occur in the absence of stimulus awareness^32^. When feedback is associated with changes in performance, it may increase the efficiency of learning, especially in difficult conditions, which has been shown, for example, in motion-direction discrimination^31^. Feedback may also promote visuomotor skill acquisition by driving learning in the motor system to better execute responses based on sensory processing^19,33–36^. Although observers may have learned to respond more reliably in the current study, the different levels of improvement across the three viewing conditions suggest that feedback had impacts on sensory processing as well.

In terms of sensory processing, feedback may impact integration of sensory information in the decision-making process during perceptual learning (e.g.^37^). Feedback may also promote learning through a recalibration in the interpretation of the available sensory cues (e.g.^38,39^) or a re-weighting of those cues according to their reliability at a given time (e.g.^21,69^,^40^,^41,42,68^). Indeed, a number of previous studies have investigated cue integration in virtual environments by manipulating cues reliabilities to change their relative weight in the resulting percept (e.g.^17,21,22^). However, in our study, we show that feedback led to the recruitment of previously-ignored sensory information. Here, we provided reliable sensory cues and found that observers were simply not using some of them in the first place, but learned to do so when explicit visual feedback was provided (see also^43^).

The improved performance observed in the feedback conditions in comparison to the no feedback conditions was not the result of greater exposure to target motion. Superior performance in the feedback conditions occurred when we equated both the number of trials and the viewing time between the feedback and no feedback conditions. Nevertheless, the finding that feedback is *required* to improve performance from very poor initial levels is important because it suggests that many individuals may require a period of training in order to get the most out of VR technology. Passive viewing of 3D media on TV or at the movies, for example, may not be a compelling and immersive experience without interactive practice with feedback. Thus, expert visual observers in the real world may be novice visual observers in the virtual world, and much like infants must learn to see in their new world^44^, so do users in the virtual world.

One of the draws of VR technology is the head tracking capability, which provides users with an interactive 360 deg view of the virtual space to enhance the types of VR experiences. In the context of virtual viewing in head-mounted displays, however, small head movements, especially involuntary ones – ‘head jitter’ - have been a concern because of the potential for mismatches between scene updates and vestibular-ocular reflex-driven eye movements to disrupt visual performance (see^45^ for a review). On the other hand, some previous work has suggested that microscopic head movements might provide reliable motion parallax information^46–48^. Although it is well-established that motion parallax information obtained through both active human and animal observer movement within an environment plays a role in depth perception (e.g.^49–53^), the extent to which motion parallax information from head jitter benefits perceptual performance has been unclear. One of the goals of the current study was to establish the conditions under which such motion parallax cues are actually used. Here we showed that when observers are free to move, but not explicitly told to do so, head jitter does benefit performance. It is noteworthy that the significant improvements in performance that occurred when head movements contributed motion parallax information were no longer observed when such motion parallax was made unreliable. This result demonstrates that in humans, like other animals, self-produced movement with concurrent visuomotor feedback is critical in the development of visually-guided behaviors^54^; however, in a VR environment, incorporating this information must be learned through explicit task feedback. Upon completion of the experiment, observers reported being subjectively unaware that they made these head movements, typically claiming that they kept their head still for the duration of the experimental blocks. Thus, the impact of head motion on sensory processing appears to be implicit with the benefit of head jitter being opportunistic. Future work will be aimed at investigating the role of head jitter in task performance and overall subjective VR experience.

A decline in performance with reduced target contrast is a common feature of performance in perceptual tasks^5,55^. The effect of target contrast in and of itself may be unsurprising, but the tendency to disproportionately report the target’s direction in depth as receding when its contrast is reduced is consistent with a heuristic of designating dimmer objects as farther away^56^. However, with task feedback, this tendency is markedly reduced as observers learn to take advantage of the additional sensory cues to motion in depth available in a virtual reality environment.

The role of target contrast is therefore an important one, especially given that accurate processing of the depth component of object motion is critical for many real world and virtual applications (crossing the street, ball catching, avoiding zombies, etc.). With the recent drive to optimize VR technology for a variety of applications from academic research to healthcare to gaming and entertainment, the new methods described here can contribute to the design and development of novel VR tasks and displays for these purposes by evaluating the expected benefit of increased reliability of a variety of sensory cues. We propose that direction misreports may be exploited as a diagnostic tool for evaluating how good a display or setup is in conveying reliable, “user-friendly” sensory information and for predicting how likely people are to achieve the goals established for the particular task and viewing environment “out of the box”.

Virtual reality allows us to bridge the gap in understanding of 3D motion perception in the natural environment, exploring the contribution of sensory signals present in the real world, but absent in traditional experimental settings while maintaining tight experimental control. Advances in affordable VR technology have set the stage to address previously intractable questions concerning human visual processing in the natural environment while maintaining precise control over experimental stimuli (see^57^ for a review). One practical concern, however, is the fidelity of the viewing experience in the virtual environment compared to that of the natural environment. That observer performance on the 3D Pong task improved *only* when feedback was provided - carrying out many trials without feedback was not sufficient for learning - demonstrates that the virtual environment does not recreate real world viewing conditions. Instead, users must learn which cues to recruit within the virtual environment.

A second major concern for VR is motion sickness^58–62,67^. Critically, recent work has shown that individuals with greater sensitivity to cues to motion in depth are more likely to experience discomfort in VR environments^63^. A proposed culprit is an enhanced ability in these individuals’ systems to recognize situations in which the sensory estimates are in a state of irresolvable conflict. The results of the current study suggest that it may be possible to train these individuals to recalibrate their use of the sensory cues to motion in depth to reduce cue conflict and associated discomfort.

In conclusion, the current study provides new insight into the nature of the sensory processing underlying accurate perception in 3D environments, in particular the perception of objects moving in depth. We have shown that poor sensitivity, and a reliance on heuristics and prior assumptions is not an inherent feature of 3D motion perception. Observers can learn to unlock the information contained within specific sensory cues, but require explicit visual feedback to do so.

## Methods

### Observers

91 members of the University of Wisconsin-Madison community provided informed consent to participate, and 72 completed all parts of the experiment. Observers who did not complete the study either: (i) had difficulty understanding the task and/or perceiving depth in the display; or (ii) experienced discomfort while wearing glasses inside the VR head-mounted display system. The study protocol was approved by The University of Wisconsin–Madison Institutional Review Board, and all experiments were conducted in accordance with relevant guidelines and regulations. Course credits were awarded in exchange for participation.

All observers had normal or corrected-to-normal vision and were screened for intact stereovision using the RANDOT Stereotest (Stereo Optical Company, Inc., 2011). To qualify for the study, observers were required to accurately identify all of the shapes in the RANDOT Form test, to identify at least 5 out of 10 targets in the RANDOT Circle test, and to pass the suppression check.

### Apparatus

The experiment was controlled by Matlab and the Psychophysics Toolbox^64–66^ on a Macintosh computer and projected through the Oculus Rift Development Kit 2 (DK2) (www.oculusvr.com), which was calibrated using standard gamma calibration procedures. The Oculus Rift DK2 is a stereoscopic head-mounted virtual reality system with a Galaxy Note 3 display - a 14.5 cm low-persistence AMOLED screen - embedded in the headset providing a resolution of 1920 × 1080 pixels (960 × 1080 pixels per eye) with a refresh rate of 75 Hz. The horizontal field of view is about 85 deg (100 deg diagonal). Head position was tracked with an accelerometer, gyroscope, and magnetometer with an update rate of 1000 Hz embedded in the headset with 0.05 deg precision, and position tracking via an external camera with near-infrared CMOS sensor with 0.05 mm precision. These tracking methods provided both head position and orientation. Observers used a wireless keyboard to initiate trials and make responses.

### Stimulus & Procedure

Observers were asked to indicate the direction of motion of a target sphere that moved with a constant velocity in the lateral and depth planes within the virtual environment. The stimuli were presented in the center of a virtual room (3 m in height, 3.52 m in width, and 3.6 m in depth). The virtual wall, ceiling, and floor were all mapped with different tiled textures to facilitate better judgment of distances throughout the virtual space, and the relative positions of the stimuli.

The stimuli were otherwise similar to those used in^5^. A planar surface stood in the center of the virtual room and was mapped with a 1/f noise pattern that was identical in both eyes to aid vergence. In the center of the surface was a gray circular aperture cut-out with a radius of 7.5 deg. Nonius lines were embedded within a small 1/f noise patch near the center of the aperture to facilitate fixation at the target’s starting position on each trial. All stimulus elements were anti-aliased to achieve subpixel resolution (Fig. 1b).

The observers’ location was 45 cm from the planar surface. Observers were instructed to fixate the center of the aperture. However, observers were free to make head movements.

Three viewing conditions were tested: (i) ‘fixed’ – in which the viewpoint of the scene did not update with observers’ head motion. This condition is akin to the traditional laboratory setup in which the head position is fixed in order to maintain a constant view of the stimulus display. (ii) ‘active’ – in which the viewpoint of the scene immediately updated in response to head motion. Diagnostic rendering tests revealed a small inherent motion-to-photon latency of 14 ms for the experimental setup. (iii) ‘lagged’ – in which the viewpoint of the scene updated in response to head motion with a constant 50 ms delay (beyond the inherent latency of the system) in one experimental version and with a random delay chosen uniformly from 0–500 ms on each trial in a second experimental version. All observers completed all three viewing conditions in a randomized, counterbalanced order. We note that in all cases, observers were free to make head movements, but they were never told explicitly to do so.

On each trial, a white sphere (‘target”) of size 0.25° in radius appeared at the center of the aperture. For 1 s, the target followed a trajectory defined by independently chosen random speeds in the x (lateral) direction and the z (motion-in-depth) direction, with no change in y (vertical direction) before disappearing. The x and z velocities were independently chosen from a 2D Gaussian distribution (M = 0 cm/s, SD = 2 cm/s) with imposed cut offs at 6.1 cm/s and −6.1 cm/s. The independently chosen speeds resulted in motion trajectories whose directions spanned the full 360° space (Fig. 1a). Thus, the target came toward the observer (‘approaching’), and moved back behind fixation away from the observer (‘receding’) on approximately 50% of trials each. It is important to note that since x- and z- motion were chosen randomly and independently, the amount of perceived lateral movement on each trial did not carry information about the amount of motion-in-depth and vice versa. The target was rendered under perspective projection, so that both monocular (looming) and binocular cues to motion-in-depth were present.

Observers indicated the direction of the target’s trajectory using a “3D pong” response paradigm^5^. After the target disappeared, a 3D rectangular block (‘paddle’), whose faces also consisted of a 1/f noise pattern, appeared at the edge of the aperture. The paddle dimensions were 0.5 cm × 1 cm × 0.5 cm. Observers were asked to extrapolate the target’s trajectory and adjust the paddle’s position such that the paddle would have intercepted the target if the target had continued along its trajectory. Observers were instructed to ignore time and only ensure the accuracy of the *location* of their response. The paddle’s position could be adjusted along a circular path that orbited the fixation point in the x-z plane using the left and right arrow keys of the keyboard (Fig. 1a). Thus, the stimuli were presented and the responses were made in the same 3D space. By asking observers to extrapolate the trajectory, we prevented observers from setting the paddle to a screen location that simply covered the last seen target location. We did not ask observers to retain fixation during the paddle adjustment phase of the trial. When the observer was satisfied with the paddle setting, they resumed fixation and pressed the spacebar.

In one version of the experiment - ‘no feedback’ - pressing the spacebar terminated the trial and initiated a new trial (n = 38). In a second version of the experiment - ‘visual feedback’ - pressing the spacebar locked in the observer’s paddle setting and the target reappeared at its last visible location and continued along its trajectory to the paddle’s path (n = 24). If the paddle intercepted the target, observers would view the interception and receive a hit (cowbell) tone; otherwise, observers would view the correct location and hear a miss (swish) tone. Pressing the up arrow key then initiated the next trial when the observer was ready. Finally, in a third version of the experiment - ‘auditory-only feedback’ - observers (n = 10) were only provided with a hit (cowbell) or miss (swish) tone upon locking in their paddle settings.

As a final manipulation, the target was presented at one of three Weber contrast levels: 0.75 (high), 0.55 (mid), and 0.47 (low), which were counterbalanced and presented in pseudorandom order.

Observers carried out 10–15 practice trials in the presence of the experimenter to become familiar with the task. All observers completed the experimental trials in one session. No feedback was provided for the practice trials. Observers in the ‘no feedback’ and ‘auditory-only feedback’ versions of the experiment completed 675 experimental trials. Because the visual feedback lengthens the time of each trial, and because we wished to prevent prolonged use of the VR headset to ensure immersion and comfort, observers in the ‘visual feedback’ version of the experiment completed 360 experimental trials.

### Data Analysis

The primary measure of performance considered here was the percentage of trials in which the paddle intercepted the target. In order to be a target interception, the paddle setting, which corresponds to the midpoint of the set paddle location must have been within 8 deg of the target’s location along the paddle’s circular path. This criterion means that some portion of the paddle made contact with the target. The percentage of trials in which the direction of the target’s motion in depth was misreported was also considered when drawing comparisons with previous work^5^.

To quantify differences across conditions, repeated-measures ANOVAs and paired-sample t-tests were used for performance within the ‘no feedback’, ‘visual feedback’, and ‘auditory-only feedback’ groups, and ANOVAs and t-tests were used for performance across the two groups. All follow-up tests were carried out with the Bonferroni-corrected alpha level for multiple comparisons.

Learning was measured by fitting an exponential function of the form f(x) = a*exp(bx) to performance over time using the Matlab fit function. Significant slope (b) parameters indicate significant changes in performance over time, reflecting learning during task exposure.

To quantify head jitter during the target’s one-second motion trajectory, we calculated the 3D head jitter on each trial (i.e., the total translation of the head in the lateral (x), vertical (y), and depth (z) directions). We summed the Euclidean distance between the head position recorded at every two adjacent time points (recorded at the screen refresh rate of 75 Hz) during the course of the target’s trajectory. To eliminate random noise in head tracking, implausible distances between two time points larger than 0.2 cm were excluded (~3.3% of all time points) when computing the head excursion. Because within-subject head jitter distributions across trials tended to be positively-skewed, we computed the median head displacement for each observer, and then averaged the sample of median head displacements to compute the between-subject magnitude of head jitter in each viewing condition (see Fig. 5a). To quantify changes in head jitter over time, linear fits were carried out on the between-subject medians for each trial (see Fig. 5b), as the between-subject head jitter distributions within trials also tended to be positively-skewed. Finally, we compared the net direction of the lateral (x) translation of the head jitter on each trial to the target’s lateral direction to characterize head jitter as moving with (same lateral direction) or against (opposite lateral direction) of target’s motion (Fig. 5c,d).
